# Patient and facility characteristics of an NDM-producing *Acinetobacter baumannii* outbreak in California, 2020–2022

**DOI:** 10.1017/ash.2023.240

**Published:** 2023-09-29

**Authors:** Lian Hsiao, Sam Horwich-Scholefield, Tisha Mitsunaga, Diana Holden, Erin Epson

## Abstract

**Background:** Carbapenem-resistant *Acinetobacter baumannii* (CRAB) are bacteria that cause healthcare-associated infections and outbreaks. Most produce carbapenemases like New Delhi metallo-β-lactamase (NDM), which are more commonly found in carbapenem-resistant Enterobacterales but rarely in CRAB. In 2018, selected laboratories began participating in a public health sentinel surveillance program by routinely submitting CRAB and other antimicrobial-resistant isolates to the AR Laboratory Network for specialized testing. In May 2020, the Antimicrobial Resistance Laboratory Network detected the first NDM-CRAB case in California, triggering an investigation. Initial whole-genome sequencing of subsequent isolates indicated high relatedness. **Methods:** We defined confirmed cases as patients with NDM detected in CRAB isolates and probable cases as NDM detected in a screening swab from a patient epidemiologically linked to a known case(s) with specimens collected during May 2020–September 2022. We defined outbreak facilities as having (1) 1 or more newly identified cases during a point-prevalence survey in response to a known case or (2) at least 2 cases identified within 4 weeks of each other that were epidemiologically linked. We analyzed demographic and specimen characteristics, as well as healthcare exposure history using R Studio version 1.3.959 software. **Results:** Of 230 total patients, 176 (77%) were confirmed and 54 (23%) were probable cases; 150 (65%) were identified through colonization screening. Among 176 NDM-CRAB isolates, the most common specimen sources were respiratory (n = 29), wound (n = 28), and urine (n = 24), and 87 (49%) of 176 isolates were nonsusceptible to all antimicrobials tested. Among patients, median age was 65 years (range, 24–97), 127 (55%) were male, 37 (15%) were Hispanic or Latino, and 100 (43%) were White. We identified 37 outbreak facilities across 13 counties, including 25 acute-care hospitals (ACHs), 6 skilled nursing facilities (SNFs), 5 ventilator-equipped SNFs (vSNFs), and 1 long-term ACH. We identified 125 cases (54%) in SNFs and vSNFs and 93 cases (40%) in ACHs; among ACH patients, 43 (46%) had been SNF or vSNF residents within the prior year. No patients reported international exposure. **Conclusions:** The first known case of NDM-CRAB in California was detected by sentinel surveillance. In this extensive regional outbreak, most cases were identified by screening at public health and clinical laboratories. Transmission occurred across healthcare settings connected by patient sharing, underscoring the importance of communication, active surveillance, and implementation of infection prevention and control practices to mitigate spread within and between facilities. Expanding these efforts, with support and resources from public health departments, is key to detecting, characterizing, and containing future outbreaks of antimicrobial-resistant pathogens.

**Figure:**
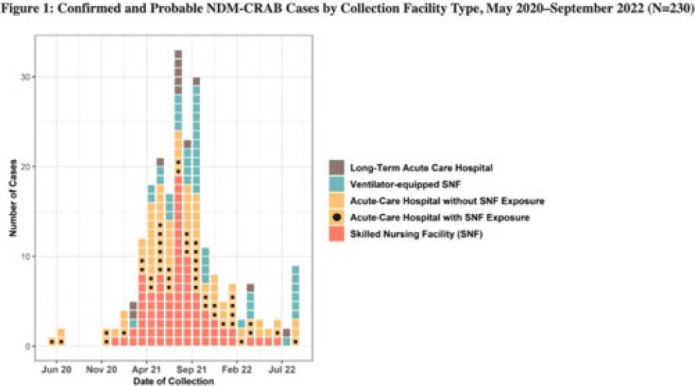

**Disclosure:** None

